# Diffusion tensor imaging of sequential neuropathological patterns in progressive supranuclear palsy

**DOI:** 10.3389/fnagi.2025.1569302

**Published:** 2025-06-06

**Authors:** Lavinia A. Bârlescu, Günter U. Höglinger, Heiko Volkmann, Albert C. Ludolph, Kelly Del Tredici, Heiko Braak, the DESCRIBE-PSP Study Group, Moritz Brandt, Hans-Peter Müller, Jan Kassubek

**Affiliations:** Department of Neurology, University Hospital Carl Gustav Carus, Technische Universität Dresden, Dresden, Germany; Institute for Stroke and Dementia Research, University Hospital, LMU Munich, Munich, Germany; Institute of Cognitive Neurology and Dementia Research, Otto-von-Guericke University, Magdeburg, Germany; Department of Neurology, University Hospital Carl Gustav Carus, Technische Universität Dresden, Dresden, Germany; Department of Neurology, University Medicine Greifswald, Greifswald, Germany; Clinic for Neurology, Medical Faculty, University Hospital Magdeburg, Magdeburg, Germany; Department of Neurology, University Hospital, LMU Munich, Munich, Germany; Institute for Stroke and Dementia Research, University Hospital, LMU Munich, Munich, Germany; Department of Neurology, University Hospital of Munich, Ludwig-Maximilians-Universität (LMU) Munich, Munich, Germany; Department of Psychosomatic Medicine, Rostock University Medical Center, Rostock, Germany; Department of Neurology, University of Bonn, Bonn, Germany; Department of Neurology, University Hospital of Munich, Ludwig-Maximilians-Universität (LMU) Munich, Munich, Germany; Charité – Universitätsmedizin Berlin, corporate member of Freie Universität Berlin and Humboldt-Universität zu Berlin-Institute of Psychiatry and Psychotherapy; Department of Psychiatry and Psychotherapy, Charité, Berlin, Germany; Department of Neurology, University Medical Centre, Rostock, Germany; Charité – Universitätsmedizin Berlin, corporate member of Freie Universität Berlin and Humboldt-Universität zu Berlin-Institute of Psychiatry and Psychotherapy; Department of Neurology, University of Bonn, Bonn, Germany; Department of Psychiatry and Psychotherapy, Charité, Berlin, Germany; Department of Neurodegenerative Diseases, Hertie Institute for Clinical Brain Research and Center of Neurology, University of Tübingen, Tübingen, Germany; Department of Psychosomatic Medicine, Rostock University Medical Center, Rostock, Germany; Department of Neurodegenerative Diseases, Hertie Institute for Clinical Brain Research and Center of Neurology, University of Tübingen, Tübingen, Germany; ^1^Department of Neurology, University Hospital Ulm, Ulm, Germany; ^2^Department of Neurology, LMU University Hospital, Ludwig-Maximilians-Universität (LMU), Munich, Germany; ^3^German Center for Neurodegenerative Diseases (DZNE), Munich, Germany; ^4^Munich Cluster for Systems Neurology (SyNergy), Munich, Germany; ^5^German Center for Neurodegenerative Diseases (DZNE), Ulm, Germany; ^6^Clinical Neuroanatomy, Department of Neurology, University of Ulm, Ulm, Germany

**Keywords:** progressive supranuclear palsy, sequential pattern, diffusion tensor imaging (DTI), neuropathology, tau protein

## Abstract

**Background and objective:**

A neuropathological cerebral staging concept for progressive supranuclear palsy (PSP) has been proposed that tau inclusions in PSP may progress in a sequential regional pattern. The objective was to develop a hypothesis-guided region/tract of interest-based (ROI/TOI) approach to use diffusion tensor imaging (DTI) targeted to analyze *in vivo* the regions that are prone to be involved at each neuropathological stage of PSP.

**Methods:**

Two data cohorts were analyzed: cohort A of 78 PSP patients [55 Richardson’s syndrome (PSP-RS) and 23 PSP with predominant parkinsonism (PSP-P)] and 63 controls, recorded at 3.0*T* at multiple sites, and a single-site cohort B constituted by 1.5*T* data of 66 PSP patients (46 PSP-RS and 20 PSP-P) and 44 controls. In cohort A, 21 PSP patients (13 PSP-RS and 8 PSP-P) and 17 controls obtained a follow-up scan after 17 months. Whole brain-based spatial statistics (WBSS) was used to identify the alterations in PSP patients vs. controls. The combined ROI- and TOI-based approach targeted structures that are prone to be involved during the course of PSP.

**Results:**

WBSS demonstrated alterations predominantly in brainstem/midbrain, basal ganglia, and frontal lobe, more pronounced in the longitudinal data. Statistical analyses of the ROIs/TOIs showed a sequential pattern of structures that were assigned to previously defined neuropathological steps.

**Conclusion:**

The combined ROI- and TOI-based DTI approach was able to map the disease stages of PSP *in vivo* cross-sectionally and longitudinally, lending support to DTI as a technical marker for imaging disease progression according to PSP stages. This approach might be useful as a tool for stratification of PSP patients MRI with respect to its proposed neuropathological progression in future longitudinal and autopsy-controlled studies.

## Introduction

Progressive supranuclear palsy (PSP), a 4R-tauopathy characterized by subcortical tau inclusions in neurons, astrocytes, and oligodendroglia, is associated with various clinical phenotypes (Kovacs et al., 2020; Höglinger et al., 2017a; Shi et al., 2021; Stamelou et al., 2021). The “classical” phenotype, Richardson’s syndrome, presents with supranuclear gaze palsy, postural instability, pseudobulbar palsy, and frontal cognitive dysfunction (Steele et al., 1964; Litvan et al., 1996). Further PSP phenotypes have been identified which differ in terms of clinical symptoms, the intensity of their manifestation and the course of their onset (Respondek et al., 2017). The phenotypes Richardson’s syndrome (PSP-RS) and predominant Parkinsonism (PSP-P) occur most frequently (Höglinger et al., 2017a; Respondek et al., 2017). To capture the multifaceted phenotypical presentations, four functional domains (ocular motor dysfunction, postural instability, akinesia, cognitive dysfunction) are defined as clinical predictors in the current MDS diagnostic criteria (Höglinger et al., 2017a). Within each domain, three clinical features contribute different levels of diagnostic certainty (probable, possible, suggestive). Neuropathologically, sequences of PSP-related tau pathology are stratified for accumulation of neuronal, astroglial, and oligodendroglial abnormal tau (Kovacs et al., 2020) into six steps for *postmortem* diagnosis: step 1 is characterized by intraneuronal tau inclusions in the globus pallidus, subthalamic nucleus, and substantia nigra; step 2 by accumulation of intraneuronal tau in the midbrain tegmentum, medulla oblongata, and pontine base, and astroglial tau pathology in the striatum. Step 3 is marked by abnormal tau inclusions in the striatum, dentate nucleus, and amygdala; step 4 by increased intraneuronal tau in the frontal lobe, whereas astroglial tau accumulates in the amygdala, parietal, and temporal lobes. Step 5 is marked by intraneuronal tau pathology in the parietal and temporal lobes, and step 6 by the increase of intraneuronal tau in the occipital cortex, whereas astroglial tau accumulates in the brainstem, globus pallidus, and cerebellar dentate nucleus. This distribution pattern concept was validated in a pathology study by Briggs and colleagues (Briggs et al., 2021).

Magnetic resonance imaging (MRI) signs of PSP have been assigned certain levels of utility in diagnostic workups and in progression monitoring (Whitwell et al., 2017) and include prominent midbrain and superior cerebellar peduncle (SCP) atrophy. Volumetric/morphometric MRI studies show frontal atrophy in PSP (Whitwell et al., 2017; Quattrone et al., 2018; Shoeibi et al., 2019; Quattrone et al., 2024) and tissue loss in fronto-/mesiotemporal cortices and prefrontal regions in addition to the central midbrain and basal ganglia (Agosta et al., 2010). Diffusion tensor imaging (DTI) is a robust MRI tool for *in vivo* investigations of white matter (WM) neuronal tracts via fractional anisotropy (FA) mapping (Basser and Pierpaoli, 1996), combined with reconstruction of fiber pathways (Mori and van Zijl, 2002). In applications to PSP, micro-structural WM abnormalities have been shown by DTI in the superior cerebellar peduncle, corpus callosum, internal capsule, pons, and basal ganglia structures (Whitwell et al., 2017; Agosta et al., 2010; Seppi and Poewe, 2010; Knake et al., 2010; Bârlescu et al., 2021). To monitor disease progression in PSP, longitudinal studies investigated MRI abnormalities of involved structures, mostly the midbrain (Quattrone et al., 2018; Höglinger et al., 2017b); Whitwell et al., 2019; Quattrone et al., 2020; Kannenberg et al., 2021) and frontal lobe. Progression of WM alterations associated with the progression of the clinical symptoms have been shown both for PSP-RS and PSP-P (Agosta et al., 2018; Caso et al., 2018).

The question arises whether longitudinal MRI changes correlate with the neuropathological progression scheme proposed by Kovacs and colleagues (Kovacs et al., 2020). The aim of the current study was the transformation of neuropathologically-defined patterns into a neuroimaging concept for *in vivo* application to individual patients. First, axonal loss and myelin degradation were mapped by unbiased voxelwise FA analysis and, second, tract-wise analysis of FA values, i.e., reconstruction of tracts-of-interest (TOI), was used for tract-specific analysis of WM integrity along disease-specific tracts (Müller et al., 2007b). In a second analysis step, a hypothesis-based analysis of DTI data, guided by the neuropathological pattern (Kovacs et al., 2020), was used to map microstructural correlates of abnormal protein propagation in the brain.

## Materials and methods

### Subjects and patient characteristics

The analysis included two cohorts of which cohort B constituted the validation sample for cohort A’s cross-sectional results. The two cohorts of PSP patients (only including the most common subtypes PSP-RS and PSP-P) fulfilled MDS diagnostic criteria (Höglinger et al., 2017a; Table 1). All patients had a certainty level of probable PSP according to the MDS criteria (Höglinger et al., 2017a) with a combination of clinical features with high specificity. Cohort A included 78 PSP patients (mean age 70.0 ± 7.4 years, male/female = 38/40); of those, 21 PSP patients underwent a follow–up scan after 17.5 months on average (range 11.3–34.8 months), and 63 controls (mean age 68.6 ± 7.7 years, male/female = 30/33, with 17 controls undergoing a follow–up scan after 17.0 months on average (range 10.0–37.4 months). Data were recorded at 3.0T from the DESCRIBE and DANCER studies of the German Center for Neurodegenerative Diseases at twelve sites (Supplementary information I); all centers used tomographs with 3.0T from the identical vendor and had implemented the identical acquisition protocol for all subjects. Cohort B included a single-site data-set of 66 patients (70.5 ± 9.1 years, male/female = 38/28) and 44 healthy controls (68.5 ± 5.3 years, male/female = 25/19), recorded at 1.5T at Ulm University. PSP patients and controls were age- and sex-matched, there were no statistical differences at baseline as well as at follow-up (see Table 1).

**TABLE 1:** Subjects characteristics.

	Cohort A (3.0T)		Cohort B (1.5T)	
	**PSP patients**	**Controls**	**PSP patients**	**Controls**
N (m/f) baseline	78 (38/40)	63 (30/33)	66 (38/28)	44 (25/19)
N (m/f) follow-up	21 (10/11)	17 (6/11)	−	−
Time to follow-up/months	17.8 ± 7.2 (11.3-34.8)	17.0 ± 7.5 (10.0-37.4)	−	−
Age (baseline)/years	70.0 ± 7.4 (50.6-86.2)	68.6 ± 7.7 (51.2-89.4)	70.5 ± 9.1 (49.0-91.3)	68.5 ± 5.3 (57.2-81.9)
Age (follow-up)/years	70.8 ± 6.5 (59.4-81.4)	71.1 ± 8.7 (53.1-82.2)	−	−
Disease duration (baseline)/years	4.2 ± 2.7 (0.5-14.4)	−	3.1 ± 1.8 (0.5-8.7)	−
Disease duration (follow-up)/years	5.2 ± 2.7 (2.4-9.8)	−	−	−
PSPRS (*a) (baseline)/points	36 ± 8 (21-52)	−	35 ± 11 (15-61)	−
PSPRS (*a) (follow-up)/points	43 ± 17 (14-70)	−	−	−
Ratio PSP-RS/PSP-P phenotype (*b) (baseline)	55/23	−	46/20	−
Ratio PSP-RS/PSP-P phenotype (*b) (follow-up)	13/8	−	−	−
Golbe (*c) stage (baseline)	2 ± 2 (1-4)	−	2 ± 1 (1-4)	
Golbe (*c) stage (follow-up)	2 ± 1 (1-3)	−	−	

PSP, Progressive supranuclear palsy; PSPRS, Progressive supranuclear palsy rating scale; PSP-RS, Progressive supranuclear palsy with Richardson’s syndrome; PSP-P, Progressive supranuclear palsy with predominant parkinsonism. (*a) Golbe and Ohman-Strickland, 2007; (*b) Höglinger et al., 2017a; (*c) Golbe et al., 2020. Values are given in mean ± standard deviation (range).

Patients were characterized by disease severity [PSP Rating Scale (PSPRS); Golbe and Ohman-Strickland, 2007], clinical stage (Golbe et al., 2020), and phenotype according to MDS Diagnostic Criteria of PSP and the MAX rules (Höglinger et al., 2017a; Grimm et al., 2019). Only PSP patients who underwent MRI scans without relevant artifacts and without imaging abnormalities compromising the accurate assessment of the scans (e.g., extended vascular lesions) were considered for the study.

This retrospective study was conducted in compliance with the declaration of Helsinki and its later amendments. All subjects provided written informed consent according to institutional guidelines approved by the DZNE Ethics Committee (DESCRIBE, Deep Phenotyping of PSP) for cohort A (reference 311/14), and Ulm University Ethics Committee for cohort B (reference 279/19).

### DTI acquisition

DTI scanning was performed by use of the following protocols: first, multicentric data (cohort A) were acquired at 3.0T scanners with 70 gradient directions (GD), including ten *b* = 0, 72 slices, 2.0 × 2.0 × 2.0 mm^3^ (matrix 120 × 120 × 72), TE = 88 ms, TR = 12,100 ms, 30 GD with b = 700 s/mm^2^; 30 GD with b = 1,000 s/mm^2^. This protocol was identical for all scanners used in the multicentric study due to the prospective planning of the data acquisition at all sites. Second, cohort B, recorded at a 1.5 Tesla scanner (Magnetom Symphony, Siemens Medical, Erlangen, Germany), contained 52 GD, including four *b* = 0, 64 slices, 2.0 × 2.0 × 2.8 mm^3^ (matrix 128 × 128 × 72), TE = 95 ms, TR = 8,000 ms, b = 1,000 s/mm^2^.

### Methodological concept

A combination of data-driven and hypothesis-guided approaches was applied: first, an unbiased voxelwise approach (whole brain-based spatial statistics, WBSS) was used to map axonal loss and myelin degradation and, second, hypothesis-guided region-of-interest (ROI) and tract-of-interest (TOI) analysis was used for analysis of WM integrity along disease-specific tracts; in a second analysis step, a hypothesis-based analysis of DTI data, guided by the neuropathological pattern (Kovacs et al., 2020), was used to map microstructural correlates of abnormal protein propagation in the brain.

### Data analysis: pre- and post-processing

Pre- and postprocessing was performed using the analysis software *Tensor Imaging and Fiber Tracking* (*TIFT*) (Müller et al., 2007a). All data were assessed for completeness, and—according to an established quality control protocol (Müller et al., 2014)

—data-sets with corrupted GD or motion artifacts were excluded from further analysis prior to correction of eddy current-induced geometric distortions. Data were transferred onto a 1 mm iso-grid for all further analyses (Müller et al., 2013). Non-linear spatial normalization to the Montreal Neurological Institute (MNI) stereotaxic standard space (Brett et al., 2002) was performed using study-specific templates and preserving directional information (Müller et al., 2007a; Andersson et al., 2003). This procedure was performed for baseline data (for subjects with only a baseline scan) and for baseline and follow-up data (for subjects with follow-up scans) using a prior intra-subject alignment (Menke et al., 2014). Fractional anisotropy (FA) maps were calculated from MNI-normalized DTI data, and a Gaussian smoothing filter of 8 mm full-width at half-maximum was applied to normalized individual FA maps (Unrath et al., 2010), providing a good balance between sensitivity and specificity. Finally, resulting FA maps were corrected for age (Behler et al., 2021). For both cohorts, a WBSS analysis was performed at the group level (see the following section “Data analysis: whole brain-based spatial statistics”), comparing males vs. females for PSP patients and for controls, respectively. No sex-dependency of FA values could be detected, thus, no correction for sex was performed in the subsequent analyses.

Briefly, PSP-RS and PSP-P display a similar sequential disease course in involved brain structures, although PSP-RS is more severe (Kovacs et al., 2020). For statistical reasons of sample size, no distinction was made in the following longitudinal analysis between PSP-RS and PSP-P, i.e., both subtypes were analyzed as one group. In order to acknowledge that progression rates are different in PSP-RS and PSP-P (Street et al., 2021), the cross-sectional comparison of PSP-RS vs. controls and PSP-P vs. controls was performed. In order to acknowledge that progression rates are different in PSP-RS and PSP-P (Street et al., 2021), the cross-sectional comparison of PSP-RS vs. controls and PSP-P vs. controls was performed.

### Data analysis: whole brain-based spatial statistics

Statistical comparisons by Student’s *t*-test were performed voxel-wise for FA values to detect changes between subject groups (whole brain-based spatial statistics, WBSS). Voxels with FA values below 0.2 were not considered for statistical comparison (Kunimatsu et al., 2004). Statistical results were corrected for multiple comparisons using the false-discovery-rate (FDR) algorithm at a significance level of *p* < 0.05 (Genovese et al., 2002). Further reduction of the alpha error was performed by a spatial correlation algorithm that eliminated isolated voxels or small isolated groups of voxels in the size-range of the smoothing kernel, leading to a threshold cluster size of 512 voxels (Müller et al., 2013); that way, only relevant structures that show statistically significant alterations survive the correction for multiple comparisons (Müller et al., 2012).

### Data analysis: tract of interest analysis

An averaged DTI data-set was calculated from the controls’ data-sets by arithmetic averaging of the MNI transformed data and was then used to identify tracts and pathways by fiber tracking (FT) for defined brain structures with a seed-to-target approach. For FT, seed and target regions were defined; all potential tracts originating in the seed region and ending in the target region (Eigenvector scalar product threshold, 0.9) of a given pathway define the corresponding TOI. As FT technique, a deterministic streamline tracking approach was used (Müller et al., 2007b). FA values within a given ROI were arithmetically averaged for each subject. The technique of tract-wise fractional anisotropy statistics (TFAS) was applied to quantify tractography results: by using the TOI, for each subject, FA values underlying the tracts were selected for arithmetic averaging. Region of interest (ROI) analysis was performed by arithmetically averaging FA values within a given ROI for each subject, considering only voxels with a FA value higher than 0.2 (Kunimatsu et al., 2004).

Student’s *t*-test (in case of normal distribution) or Mann-Whitney-U-test (in case of not normal distribution) were used to compare ROI- and TOI-based FA values statistically at the group level. Significance was defined at *p* < 0.05, FDR-corrected for multiple comparisons. Supplementary information II summarizes localization and size of the ROIs/TOIs studied.

### ROI/TOI definitions

**FIGURE 1:**
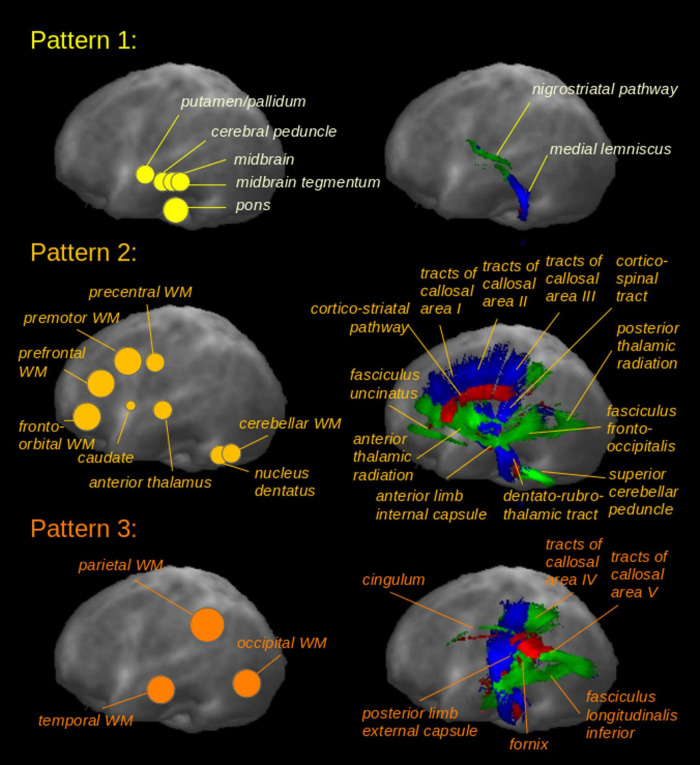
Regions-of-interest (ROIs) and tracts-of-interest (TOIs) selected for PSP patterns 1-3 based on previous findings in imaging and neuropathological studies. Sagittal projectional ROI (left panel) and TOI (right panel) representations for the three patterns corresponding to sequential involvement of brain structures during the course of PSP.

Multiple brain structures known from imaging and neuropathological studies to be affected in PSP were analyzed. The structures were divided into three phases of disease progression. The early phase (pattern 1) corresponds to steps 1-2 of the sequential neuropathological involvement described by Kovacs and colleagues (Kovacs et al., 2020); the intermediate (pattern 2) and late (pattern 3) phases correspond to steps 3-4 and steps 5-6, respectively (Table 2). Figure 1 illustrates the ROIs/TOIs which were assigned to the three PSP patterns.

**TABLE 2:** ROIs and TOIs selected for PSP phases 1-3 based on previous findings in imaging and neuropathological studies.

Phase (pattern)	Region or tract	Imaging studies	Neuropathology studies
**Early phase** **(pattern 1)**	**Brainstem and basal ganglia**		
	Putamen, globus pallidus	Whitwell et al., 2017 Armstrong, 2018 Potrusil et al., 2020	Kovacs et al., 2020 Quattrone et al., 2024 Dickson et al., 2007 Chougar et al., 2021
	Pontine tegmentum	Whitwell et al., 2017	Kovacs et al., 2020 Quattrone et al., 2024 Dickson et al., 2007
	Medial lemniscus	Worker et al., 2014	
	Nigrostriatal tract	Albrecht et al., 2019 Lee and Lee, 2019 Nguyen et al., 2021	
	Cerebral peduncles	Albrecht et al., 2019 Lee and Lee, 2019 Nguyen et al., 2021 Chougar et al., 2021	
	Subthalamopallidal tract		
**Intermediate phase (pattern 2)**	**Basal ganglia, diencephalon,** **frontal cortex white matter and white matter tracts, cerebellum**		
	Caudate nucleus	Whitwell et al., 2017 Chougar et al., 2021 Potrusil et al., 2020	Kovacs et al., 2020; Dickson et al., 2007
	Thalamocortical radiations (anterior, posterior)	Potrusil et al., 2020 Gorges et al., 2017	
	Fronto-orbital, prefrontal, premotor and precentral white matter	Whitwell et al., 2017 Quattrone et al., 2024 Brenneis et al., 2004 Paviour et al., 2006 Sakurai et al., 2017	Kovacs et al., 2020; Dickson et al., 2007
	Fronto-occipital and uncinate fascicles	Caso et al., 2018 Potrusil et al., 2020	
	Corpus callosum (areas I-III)	Bârlescu et al., 2021 Nguyen et al., 2021 Padovani et al., 2006 Rosskopf et al., 2014 Lenka et al., 2017	
	Internal capsule (anterior limb)	Spotorno et al., 2019 Nicastro et al., 2020	
	Corticostriatal tract		
	Cerebellar white matter	Whitwell et al., 2017	Kovacs et al., 2020 Robinson et al., 2020
	Cerebellar dentate nucleus	Chougar et al., 2021	Kovacs et al., 2020 Quattrone et al., 2024 Dickson et al., 2007
	Superior cerebellar peduncle	Quattrone et al., 2024 Potrusil et al., 2020 Sakurai et al., 2017 Archer et al., 2020 Whitwell et al., 2021 Quattrone et al., 2022 Canu et al., 2011	Dickson et al., 2007 Chougar et al., 2021
	Dentatorubrothalamic tract	Whitwell et al., 2017 Potrusil et al., 2020 Whitwell et al., 2011 Rau et al., 2022	Dickson et al., 2007
	Corticospinal tract	Potrusil et al., 2020 Gorges et al., 2017 Zanigni et al., 2017	
**Late phase (pattern 3)**	**Cerebral cortex and cortical white matter tracts**		
	Parietal, temporal, and occipital lobe white matter	Burciu et al., 2015 Josephs et al., 2013	Kovacs et al., 2020 Sakae et al., 2019
	Inferior longitudinal fascicle	Sarica et al., 2021 Clark et al., 2021	
	Internal capsule (posterior limb)	Sarica et al., 2021 Clark et al., 2021	
	Corpus callosum (areas IV-V)	Hofer and Frahm, 2006	
	Fornix	Whitwell et al., 2017	
	Cingulum bundle	Potrusil et al., 2020 Canu et al., 2011	

For the early phase, we selected structures of the brainstem and basal ganglia: in clinical MRI, a core feature of PSP is midbrain atrophy (Whitwell et al., 2017; Stamelou et al., 2011; Potrusil et al., 2020; Sakurai et al., 2017) which is also observed in neuropathological studies (Kovacs et al., 2020; Dickson et al., 2007). Volumetric measures of the putamen and globus pallidus are reduced (Whitwell et al., 2017; Armstrong, 2018; Potrusil et al., 2020) and tau inclusions are severe (Kovacs et al., 2020; Dickson et al., 2007). The globus pallidus is one of the earliest affected structures (Dickson et al., 2007). The pontine tegmentum is less severely affected (Whitwell et al., 2017; Dickson et al., 2007). The cerebral peduncles and nigrostriatal tract interconnect early-involved brain structures and are the subject of recent imaging studies (Albrecht et al., 2019; Lee and Lee, 2019; Nguyen et al., 2021). We included the medial lemniscus (Worker et al., 2014), owing to its course through the brainstem. For the intermediate phase, we analyzed further basal ganglia components and the diencephalon (caudate nucleus, thalamus) which exhibit microscopic changes and imaging correlates of degeneration (Kovacs et al., 2020; Whitwell et al., 2017; Dickson et al., 2007; Chougar et al., 2021; Potrusil et al., 2020; Robinson et al., 2020). Given their anatomical connections and known involvement in DTI studies in PSP (Potrusil et al., 2020; Gorges et al., 2017), we also included the thalamic radiations. Frontal WM involvement is reported by multiple studies (Kovacs et al., 2020; Whitwell et al., 2017; Quattrone et al., 2024; Dickson et al., 2007; Brenneis et al., 2004; Paviour et al., 2006; Sakurai et al., 2017); here, we divided it into four ROIs. We also assigned to this phase frontal lobe WM tracts that display abnormalities in DTI studies (Caso et al., 2018; Potrusil et al., 2020) Because neuroimaging detects corpus callosum changes, especially of the anterior part (Bârlescu et al., 2021; Nguyen et al., 2021; Padovani et al., 2006; Rosskopf et al., 2014; Lenka et al., 2017), we included its frontal-most three segments (Hofer and Frahm, 2006), together with the anterior limb of the internal capsule (Spotorno et al., 2019; Nicastro et al., 2020) that has reciprocal thalamic, striatal, and frontal lobe projections, as well as the corticostriatal tract because of its connection with the striatum. PSP imaging and histological studies (Kovacs et al., 2020; Whitwell et al., 2017; Chougar et al., 2021; Robinson et al., 2020) report cerebellar WM and dentate nucleus abnormalities; we selected these structures together with the superior cerebellar peduncle, a WM bundle used for confirming the diagnosis (Quattrone et al., 2024; Dickson et al., 2007; Chougar et al., 2021; Sakurai et al., 2017; Archer et al., 2020; Whitwell et al., 2021; Quattrone et al., 2022; Canu et al., 2011). The dentatorubrothalamic tract connects the dentate nucleus with the red nucleus and the thalamus; it was specifically chosen as a separate structure owing to reports of degenerative changes in PSP (Whitwell et al., 2017; Dickson et al., 2007; Potrusil et al., 2020; Whitwell et al., 2011; Rau et al., 2022). Finally, given its origins predominantly in the frontal cortex and its role in movement, we assigned the corticospinal tract to phase 2 (Potrusil et al., 2020; Gorges et al., 2017; Zanigni et al., 2017). In late-phase PSP, imaging and histopathological studies report degeneration of the cerebral cortex and the parietal, occipital, and temporal lobe WM (Kovacs et al., 2020; Quattrone et al., 2024; Sakae et al., 2019; Burciu et al., 2015; Josephs et al., 2013). Together with these regions, we analyzed the inferior longitudinal fascicle and internal capsule (internal limb) that involve the occipital lobe (Gorges et al., 2017; Sarica et al., 2021; Clark et al., 2021). Finally, we selected areas IV-V of the corpus callosum which connect to the parietal, occipital and temporal lobes (Hofer and Frahm, 2006) and additional WM tracts (fornix, cingulum bundle), because regional changes there are known from PSP-based imaging studies (Whitwell et al., 2017; Potrusil et al., 2020; Gorges et al., 2017; Canu et al., 2011).

### Pattern categorization and classification of PSP patients

In regions where a (highly) significant difference in FA values at the group level was identified, there is nevertheless—as an intrinsic property of the voxelwise comparison at the group level

—an overlap of the FA values of PSP and controls (see Supplementary information III for an example of overlap values). In order to take this property into account in the transition to an analysis at the individual level, a threshold value of μ−0.47σ was defined (Note: with a normal distribution, this value corresponds to a probability of 68% for controls to be above the threshold value). That way, thresholds were defined so that the specificity of each pattern reached 77.8% for cohort A and 77.3% for cohort B, i.e., 49 of 63 controls for cohort A and 34 of 44 controls for cohort B were defined as “different from PSP.” This approach was chosen in analogy to FA analyses in other neurodegenerative diseases, e.g., amyotrophic lateral sclerosis (Kassubek et al., 2014). For each ROI/TOI structure, FA values were normalized to mean and standard deviation of FA values of the controls (z-transformation) in order to obtain values comparable between different anatomical structures. Then, *z*-values for the ROI/TOI structures of each pattern were arithmetically averaged to obtain a pattern-specific < FA > value for each subject (pattern 1, 2, or 3). A threshold was defined so that specificity of each pattern was 77.8% for cohort A and 77.3% for cohort B, i.e., 49 of 63 controls for cohort A and 34 of 44 controls for cohort B were defined as “different from PSP.” This procedure was chosen in analogy to FA analyses in other neurodegenerative diseases, e.g., amyotrophic lateral sclerosis (Kassubek et al., 2014).

**FIGURE 2:**
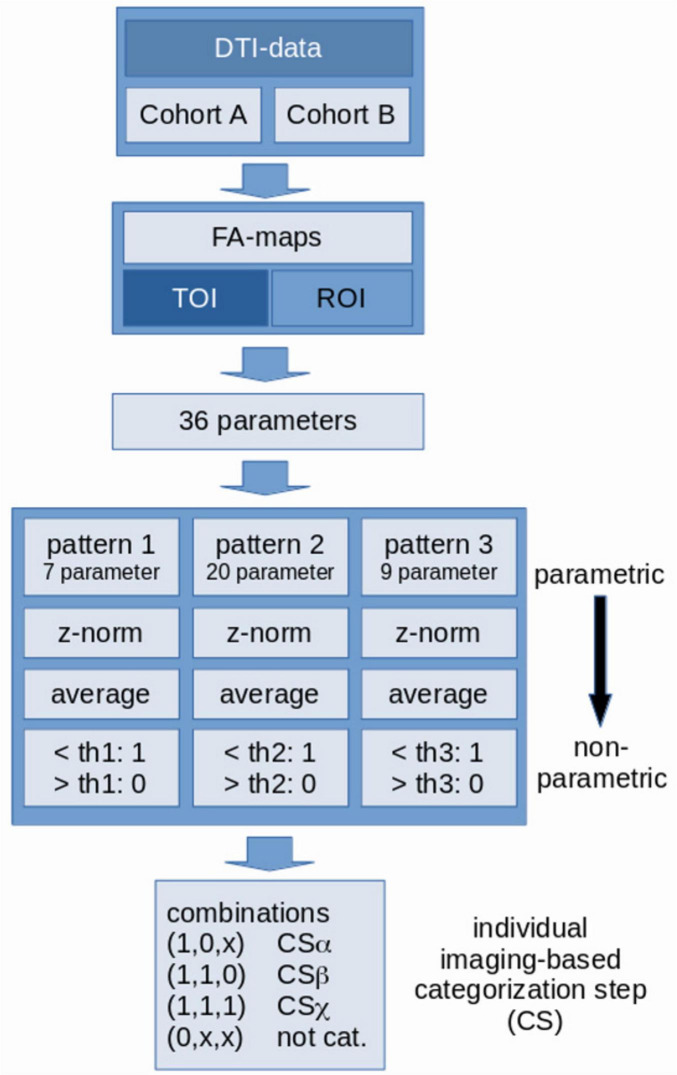
Scheme for data analysis and categorization of PSP patients. Fractional anisotropy (FA) maps were calculated from DTI data, i.e., 36 parameters were calculated for each subject from combined region-of-interest (ROI) and tract-of-interest (TOI) analyses (see Supplementary information II). Parameters were sorted into 3 patterns and for each pattern (after “z-normalization”) a threshold-based decision of affectation (1 or 0) was performed. The combinations of pattern-specific decisions [the individual imaging-based categorization step (CS)] lead to a categorization into CSα, CSβ, and CSχ.

A categorization was then performed that classified each patient into one of three categorization steps (CS) according to the neuropathological staging concept (Kovacs et al., 2020), where CSα corresponds to steps 1-2, CSβ to steps 3-4, and pattern CSχ to steps 5-6 (Kovacs et al., 2020). For PSP patients, CSα is reached when z(< FA >) in pattern 1 is below the threshold of pattern 1 and z(< FA >) in pattern 2 is above the threshold of pattern 2; CSβ is reached when z(< FA >) in pattern 1 is below the threshold of pattern 1, z (< FA >) in pattern 2 is below the threshold of pattern 2, and z(< FA >) in CSχ is above the threshold of pattern 3; CSχ is reached when z(< FA >) in all patterns is below the threshold of the respective pattern. Figure 2 provides an analysis scheme.

### Data availability

The dataset used and analyzed during the current study will be made available by the corresponding author upon reasonable request to qualified researchers.

## Results

### Unbiased whole brain-based statistics

**FIGURE 3:**
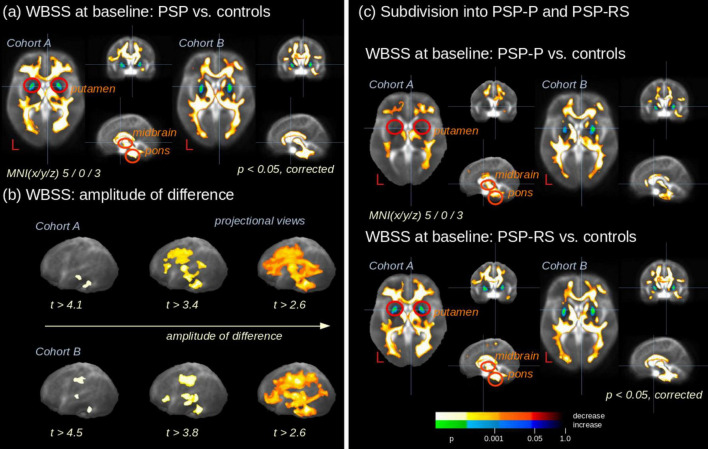
Whole brain-based spatial statistics (WBSS) of fractional anisotropy (FA) maps. **(a)** Exemplary slicewise representation of WBSS of FA maps of 78 PSP patients vs. 63 controls at baseline (cohort A) and 66 PSP patients vs. 44 controls at baseline (cohort B). A decrease of FA in PSP patients vs. controls is displayed in hot colors, an increase in cold colors. **(b)** Differences at the group level (illustrated by variation of thresholded *t*-values) in projectional sagittal views for cohorts A and B. **(c)** Subdivision of WBSS into the subtypes PSP with Richardson’s syndrome (PSP-RS) and PSP with predominant parkinsonism (PSP-P). MNI, Montreal Neurological Institute coordinate frame.

Statistical comparison of 78 PSP patients vs. 63 controls (cohort A) at baseline showed widespread alterations in multiple WM areas (Figure 3a). The comparison between the 66 patients vs. 44 controls from cohort B at baseline confirmed these results (Figure 3a). A separation into PSP-P and PSP-RS subtypes (Table 1) and separate comparison to controls demonstrated similar patterns in cohort B for both subgroups, whereas, in cohort A, PSP-RS displayed more pronounced alterations than PSP-P, especially in the putamen. In both cohorts, the PSP-RS variant was significantly more affected than PSP-P (Figure 3c and Supplementary information IV).

Variation of the *t*-value threshold showed a pattern of significant clusters that reminded of the hypothesized spreading concept of PSP: alterations with the highest *t*-values were observed in the midbrain and premotor WM, moderate alterations were observed in structures assigned to pattern 2, and the additional alterations at more lenient thresholds were observed in the parietal, temporal, and occipital WM (pattern 3) (Figure 3b and Supplementary information IV). This pattern was the same for cohorts A and B. A difference for PSP-P vs. controls existed in the putamen (Figure 3c)—this was bilaterally significant for cohort B (FA increase), whereas for cohort A, no significant putaminal alterations were found. However, closer inspection showed that, for cohort A, putaminal changes were present but disappeared in the analysis owing to the small cluster size. A direct voxelwise comparison of the two groups (PSP-RS and PSP-P patients) showed no significant result clusters for statistical reasons – the comparison of baseline and follow-up of patients vs. controls at baseline has the advantage of an improved signal to noise ratio compared to direct comparison of patient groups, for an analytical proof please refer to Müller and Kassubek (2024). In summary, cohort B is a validation sample for A’s cross-sectional results.

**FIGURE 4:**
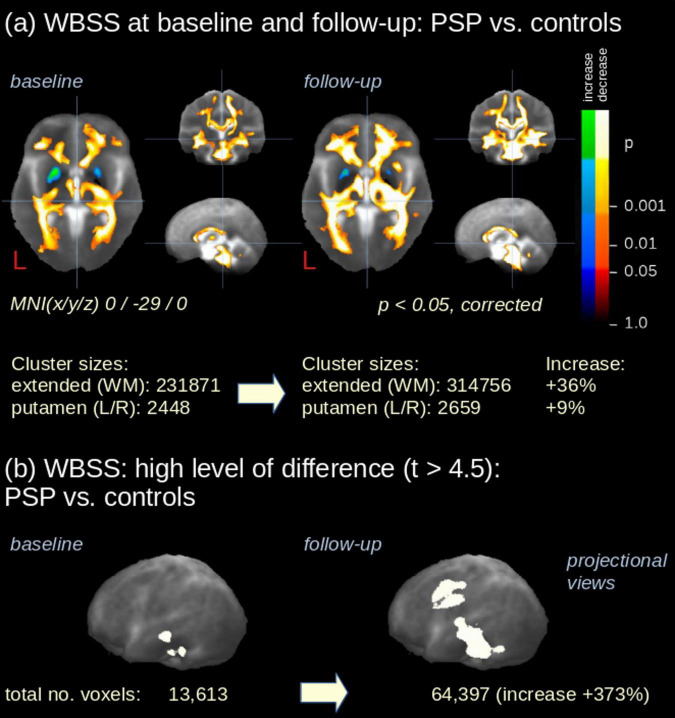
Whole brain-based spatial statistics (WBSS). **(a)** Slicewise representation of WBSS (*p* < 0.05, corrected for multiple comparisons) of FA maps of 21 PSP patients vs. 63 controls at baseline and at follow-up. **(b)** Results at a thresholded amplitude of difference (*t*-values) in projectional sagittal views for baseline and at follow-up. MNI, Montreal Neurological Institute coordinate frame.

The longitudinal data comprised the subgroup of 21 patients from cohort A who had obtained follow-up scans. Baseline and follow-up data of these patients were compared with data from 63 controls at baseline by WBSS. Baseline comparisons showed a pattern of FA alterations similar to the comparison of the whole data samples of cohorts A and B at baseline, while follow-up scans showed extended alteration clusters in the longitudinal comparison compared to baseline comparison (Supplementary information IV). At a significance level of *p* < 0.0001, baseline data showed significant midbrain clusters (together 13,613 voxels), while longitudinal data showed extensive significant clusters in midbrain and premotor and motor areas (64,397 voxels) (Figure 4), indicating an expansion of the altered structures from baseline to follow-up.

### Hypothesis-guided ROI/TOI analyses

Corresponding to anatomical localization and connectivities, selected WM tracts classified to be associated with one of the aforementioned phases (refer to Table 2 and references therein) were analyzed.

From cohort A, 17 controls received a follow-up scan. Their data at baseline and follow-up were used to calculate the variability of FA parameters; the mean differences between baseline and follow-up of all ROIs/TOIs were Δ < FA > = 0.006 ± 0.007. This value constitutes the lower limit of FA-based detectability of pathological changes. As FA maps were age-corrected, this analysis was used to determine the influence of environmental noise and other measurement-specific factors: the average differences between baseline and follow-up of all ROIs/TOIs was Δ < FA > = 0.006 ± 0.007. Supplementary information V shows differences of < FA > for ROIs/TOIs between patients and controls at baseline (for cohorts A and B), subdivided into pattern 1-related structures, pattern 2-related structures, and pattern 3-related structures, respectively. Most structures (depending on the cohort) and especially the grand average over patterns 1, 2, and 3 displayed a significant reduction of < FA > in patients compared to controls; the exception was the putamen where an increase of < FA > in patients was observed.

*Z*-values for ROI/TOI structures were arithmetically averaged for each pattern to obtain a pattern-specific < FA > value for each subject (pattern 1, 2, or 3, respectively). Table 3I summarizes the group-averaged results for the three patterns for cohorts A and B. At group level, all patterns for cohorts A and B showed significant differences; based thereon, the patients were categorized into steps CSα, CSβ, CSχ (Table 3II). Only eight cohort A patients and one cohort B patient eluded categorization. Because both cohorts showed similar results, we confirmed that cohort B could be regarded as a validation sample for the cross-sectional results from cohort A.

**TABLE 3:** Pattern analysis results for cohorts A and B and pattern categorization into steps CSα, CSβ, CSχ.

		Cohort A				Cohort B			
**(I) Pattern analysis**									
		**Pattern 1**	**Pattern 2**	**Pattern 3**		**Pattern 1**	**Pattern 2**	**Pattern 3**	
z(< ΔFA >)	Controls	0.50	0.50	0.50		0.50	0.50	0.50	
	PSP	−0.74	−0.49	−0.25		−0.81	−0.89	−0.72	
P		1e-16	1e-13	1e-7		1e-20	1e-13	1e-12	
Specificity/%		77.8	77.8	77.8		77.3	77.3	77.3	
Sensitivity/%		89.7	73.1	59.0		98.5	80.3	81.8	
**Pattern analysis for subtypes PSP-P and PSP-RS**									
z(< ΔFA >)	PSP-P	−0.65	−0.39	−0.32		−0.47	−0.82	−0.85	
P		1e-6	1e-4	1e-3		1e-4	1e-4	1e-5	
z(< ΔFA >)	PSP-RS	−0.78	−0.53	−0.23		−0.95	−0.92	−0.66	
P		1e-12	1e-11	1e-5		1e-19	1e-10	1e-8	
**Pattern analysis for PSP patients according PSPRS**									
		Pattern 1	Pattern 2	Pattern 3		Pattern 1	Pattern 2	Pattern 3	
z(< ΔFA >)	PSPRS 1	−0.51	−0.33	−0.11		−0.62	−0.75	−0.67	
	PSPRS 2	−0.82	−0.50	−0.29		−0.59	−0.63	−0.54	
	PSPRS 3 + 4	−1.19	−0.96	−0.61		−1.45	−1.52	−1.10	
**(II) Pattern categorization**									
		*CSα*	*CSβ*	*CSχ*	Not cat.	*CSα*	*CSβ*	*CSχ*	Not cat.
N (pattern)	PSP	14	14	42	8	12	3	50	1
**Pattern categorization for subtypes PSP-P and PSP-RS**									
N (pattern)	PSP-P	6	3	11	3	3	0	16	1
N (pattern)	PSP-RS	8	11	31	5	9	3	34	0
**Pattern categorization (baseline and follow up – N = 21)**									
N (pattern)	PSP (baseline)	4	3	13	1				
	PSP (follow up)	3	2	16	0				
**(III) Longitudinal alterations (cohort A)**									
		N	Pattern 1	Pattern 2	Pattern 3				
z (< ΔFA >)	contr.(baseline)	63	0.50	0.50	0.50				
	PSP (baseline)	21	−0.84	−0.67	−0.41				
	PSP(follow-up)	21	−1.21	−1.00	−0.84				
Δz (< ΔFA >) (baseline, follow-up)			−0.37	−0.33	−0.42				
p [PSP (baseline) vs. contr. (baseline)]			1e-7	4e-6	1e-4				
p [PSP (follow-up) vs. contr. (baseline)]			1e-11	6e-8	2e-5				
p [PSP (follow-up) vs. PSP (baseline)]			0.11	0.21	0.16				

(I) The pattern analysis showed arithmetically averaged *z*-values [z (< FA > ] for ROI and TOI structures, summarized into patterns 1, 2, and 3: *z*-values, *p*-values in group comparison, and specificity and sensitivity for group separation between PSP patients and controls. The difference of *z*-values for PSP patients (categorized into PSPRS groups 1, 2, and 3 + 4) to controls [z (< FA > = 0.5] increases with higher PSPRS group. (II) Pattern categorization shows the results of categorization of PSP patients into steps CSα, CSβ, CSχ and “not categorized”. (III) Longitudinal pattern analysis results for cohort A for baseline and follow-up (*N* = 21 PSP-patients). CS, imaging-based categorization step.

### Longitudinal analysis

**FIGURE 5:**
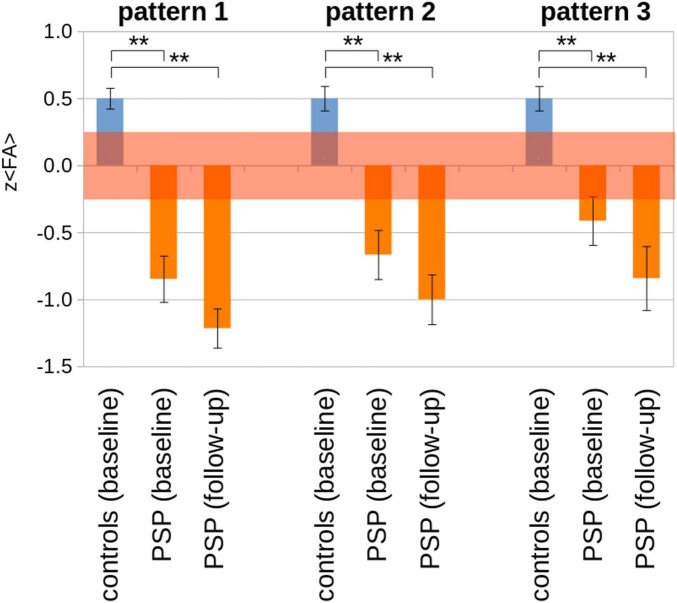
Pattern analysis results [z (< FA >)] for cohort A for baseline and follow-up (*N* = 21 PSP-patients) vs. controls (*N* = 63). The transparent red interval indicates an error estimation obtained from baseline and follow-up controls’ scans. **p < 0.0001.

Of the 21 patients in cohort A with at least one follow-up scan, seven had a substantial clinical progression (i.e., increase of the PSPRS stage). The corresponding z(< FA >) values are summarized in Table 3III and Figure 5. A significant decrease in z(< FA >) from baseline to follow-up compared to controls at baseline was observed for all patterns, and a tendency (albeit not significant) of decreased z(< FA >) was observed in patients from baseline to follow-up. Individual examples of longitudinal alterations of z(< FA >) for the three patterns are demonstrated in Supplementary information VI. As progression rates are different in PSP-RS and PSP-P (Street et al., 2021), the cross-sectional comparison of 13 PSP-RS vs. controls and eight PSP-P vs. controls (for whom a follow-up scan was available) was performed at baseline and at follow-up (although with low statistical impact due to low subject numbers), in order to analyze the extent to which corresponding brain regions were affected. The analysis PSP-RS vs. controls showed more extended significant FA decrease compared to the PSP-P vs. controls at baseline; at follow-up, both groups showed an increase of affection clusters. Due to statistical reasons (low subject numbers), FA increases in the bilateral putamen for PSP-P and in the right putamen for PSP-RS were below the detection threshold (Supplementary information VII).

### Imaging-based step categorization

Table 3II summarizes the categorization of individual subjects into PSP steps by *in vivo* ROI/TOI analysis for cohorts A and B, separated for cross-sectional and longitudinal analysis. The specificity of 77.8% (cohort A) and 77.3% (cohort B) was associated with a high sensitivity of 89.7% (cohort A) and 98.5% (cohort B), respectively. Of the total number of patients (cohorts A and B; N = 144), 93.8% could be assigned to imaging-based categorization steps CSα, CSβ, and CSχ. The categorization for the 21 patients with baseline and follow-up scans showed an increase to higher categorization steps from baseline to follow-up. Supplementary information VIII shows the percentage distribution. Effect sizes and sample sizes for the patterns 1, 2, and 3 were summarized in Supplementary information IX.

### Categorization according to the PSP rating scale

According to the PSPRS, patients were categorized into PSPRS stages 1, 2, 3 + 4; one patient from cohort A and six patients from cohort B had PSPRS stage 4, and no patient had PSPRS stage 5. WBSS of PSPRS subgroups displayed more marked alterations corresponding to more marked FA decreases from PSPRS 1 to PSPRS 3 + 4 for both cohorts (Supplementary information X). Cohort A showed the highest z(< FA >) differences from controls [z (_*controls*_ = 0.50; Table 3I] for the PSPRS 3 + 4 group in pattern 1, decreasing to pattern 3 and also decreasing to the PSPRS 1 group (thereby indicating highest FA alterations in the PSPRS 3 + 4 group for all patterns) and a decrease from pattern 1 to pattern 3 for all PSPRS groups. Cohort B had similar results; however, the FA decrease in cohort B was not as clearly pronounced with respect to the PSPRS classification as in cohort A.

## Discussion

We investigated whether DTI can track progression of PSP and if this *in vivo* imaging-based categorization follows the proposed neuropathological PSP progression concept (Kovacs et al., 2020), in order to identify potential neuroimaging correlates of PSP-associated brain pathology at the group level. Structural connectivity analysis by DTI was considered to be an excellent candidate for investigating microstructural alterations during the course of neurodegenerative diseases (Kassubek and Müller, 2020; Kassubek et al., 2018). Corresponding to the progression of PSP pathology according to Kovacs et al. (2020), a sequential pattern of regional changes was demonstrated in two independent cohorts. A combination of analysis approaches to capture these changes has been used: first, cross-sectional unbiased WBSS was applied—the highest differences at the group level (in terms of *t*-values) resulted in areas involved at disease onset, whereas lower differences at the group level corresponded to later disease stages. The logic that an association with disease stages exists depending on the alteration differences is explained by simulations by Gorges and colleagues (Gorges et al., 2018): sequential involvement is a possible model for the disease alteration pattern revealed by WBSS analysis. Second, hypothesis-guided ROI/TOI analyses showed groups of ROIs/TOIs that could be summarized into imaging-based categorization steps CSα, CSβ, CSχ which displayed sequential involvement of disease stage-related patterns at the group level for the PSP patients. Longitudinally, both the WBSS and the ROI/TOI analyses demonstrated an increase of the brain alteration pattern after a 17-month observation period. A sophisticated model (which already has been applied in the analysis of atrophy patterns in PSP) is the “Subtype and Stage Inference” (SuStaIn) model (Saito et al., 2022); this model was able to identify distinct temporal progression patterns of brain atrophy for corticobasal syndrome and PSP-RS with high reproducibility and high accuracy based on cross-sectional structural brain MRI data. Given that PSP-RS and PSP-P displayed a similar sequential disease course for involved brain structures (Kovacs et al., 2020), the two subtypes were analyzed as one group. Nevertheless, the cross-sectional comparison of PSP-RS vs. controls and PSP-P vs. controls showed fewer alterations in the PSP-P group, albeit in the same brain regions.

Longitudinal scans of controls were used to estimate the variability of FA at the group level; this estimation can serve as a “threshold” for alterations detected in PSP patients, which were significantly higher than in controls so that FA alterations in patients at the group level can be considered to be pathological. However, due to the diverse sources of variability in *in vivo* DTI scans, we reduced the six neuropathological steps to three imaging-based categorization steps (CSα, CSβ, CSχ) so that a higher degree of stability in the resulting patterns could be obtained. Furthermore, the large number of ROIs/TOIs helped to overcome DTI variability and the inhomogeneity in clinical presentation of PSP. One strength of this study was that cross-check could be performed because two independently obtained patient and control cohorts were evaluated, and the results from both cohorts displayed a high degree of agreement and consistency.

In this study, longitudinal outcomes were used as a validation to identify disease-related structures and their involvement in PSP over the course of the disease; accurate modeling of disease progression would require a significant higher amount of cross-sectional and longitudinal DTI (and structural MRI) data. Approaches to longitudinal atrophy of brain structures in PSP (Scotton et al., 2023) and tractography-associated changes in PSP (Costa et al., 2025) address disease progression and can be modeled as temporal progression patterns by current approaches such as “Subtype and Stage Inference” (SuStaIn) (Saito et al., 2022).

The 4R-tauopathy PSP is characterized by subcortical tau inclusions in neurons, astrocytes, and oligodendroglia leading to the concept of PSP-associated sequences of tau progression (Kovacs et al., 2020). WM damage measured using diffusion tensor imaging (DTI) is also a consistent feature of PSP and is thought to result from a tau-driven neurodegenerative process partially independent of gray matter degeneration (Costa et al., 2025; Armstrong, 2013). DTI and its resulting metrics address a physical parameter (diffusion) and, thus, indirectly measure a grade of integrity especially in WM tracts. Alterations in DTI metrics could result from fiber loss, synaptic loss, or loss of fiber integrity; while FA is sensitive to microstructural changes, it does not indicate a specific type of lesion. On the other hand, AD tends to be strongly affected by axonal injury whereas RD is sensitive to white matter damage due to demyelination and less to changes in the axonal density or size (Song et al., 2002; Winklewski et al., 2018; Wang et al., 2015). That way, the advantage of application of DTI as a non-invasive *in vivo* technique is shadowed by the fact that DTI metrics do only indirectly measure tau pathology and, thus, could be affected by further diffusion-influencing factors. Diffusion properties represented by the voxelwise diffusion ellipsoid could be parameterized by a series of parameters which are essentially divided into voxelwise parameters (e.g., FA, mean diffusivity, radial and axial diffusivity) and tractwise parameters (resulting from the feasibility of tract reconstruction; Schilling et al., 2021). In the first case, the voxelwise parameters result from a parameterization by different combination of the Eigenvalues of the ellipsoid; therefore, in order to avoid redundancy, it is crucial to choose one of these parameters when mapping disease-related diffusion alterations *in vivo*. In the second case, fiber tractography bundle segmentation depends on scanner effects, vendor effects, acquisition resolution, diffusion sampling scheme, and diffusion sensitization. FA is particularly suitable because of its intrinsic normalization to values between 0 and 1, which then indicate the strength of the directionality of the diffusion.

This study aimed to investigate regional DTI changes longitudinally corresponding to neuropathological stages of PSP-associated sequences of tau progression (Kovacs et al., 2020). Regions with neuropathologically proven tau inclusions or degenerative changes served as a basis for ROIs/TOIs. Some of these involved gray matter, others WM regions and tracts. Selection was based on the involvement reported in previous neuropathology or imaging studies and/or the relationship to related structures that displayed involvement (mostly WM regions and tracts) (Table 2 and Figures 3, 4). Prior research showed that brain connectivity in PSP is linked to tau deposition (Franzmeier et al., 2022) in support of DTI findings of WM damage; moreover, the possible contribution of these anatomical regions to PSP symptomatology was a rationale for their inclusion (Kovacs et al., 2020; Dickson et al., 2007). In that context, the findings in the putamen need to be commented on, given that FA decrease is caused by axonal loss or myelin degradation. Still, FA elevation in the putamen remains an unresolved challenge, as gray matter has a more isotropic diffusion profile, making DTI metrics such as FA less meaningful due to its complex cytoarchitecture. DTI metrics are influenced by iron accumulation which occurs physiologically with aging so that FA levels in the putamen and caudate have been shown to be higher in healthy older subjects (Pfefferbaum et al., 2010); however, the age correction approach (Behler et al., 2021) used in our study accounts for both negative and positive age-associated FA changes (which in parallel could occur in different brain regions) due to voxelwise correction. Structural neuroimaging of PSP already showed degeneration throughout a network of gray matter structures, including midbrain, thalamus, subthalamic nucleus, globus pallidus, striatum and frontal cortex, and especially for PSP-RS and PSP-P in the putamen (Whitwell et al., 2020; Spiegel et al., 2025); furthermore, among further tracts, the corticostriatal tract showed progression in the course of disease (Costa et al., 2025). However, as tau pathology is not the only underlying pathological mechanisms (synaptic dysfunction and loss may precede tau accumulation have to be considered), the DTI-based results of the current study have to be regarded as an indicator for putaminal involvement and require additional confirmation by future studies. The FA increase pattern detected in the putamen most probably resulted from pathological processes that modify tissue integrity, e.g., neuronal remodeling and loss, secondary astrocytosis, loss of specific fiber tracts, alterations in fiber composition, or changes in cell permeability (Rosas et al., 2006; Douaud et al., 2009; Douaud et al., 2011). Therefore, a specifically pronounced loss of fiber integrity/directionality might result in an apparent FA increase.

Our findings must be considered within the context of several limitations. The challenges of *in vivo* DTI in neurodegeneration are manifold: (1) different sources of variability may contribute to the diffusion data of subjects, e.g., scanner variability, environmental noise, individual subject factors, which cannot be singled out but may bias the data (Müller et al., 2013). We are aware that there was an imbalance of participants (PSP and controls) of cohort A across the scanners (Supplementary information I) which may impact the group comparisons. However, given that the identical acquisition protocol had been implemented on all scanners (all of the same vendor) and we have additionally tested for the inter subject-homogeneity of whole-brain FA values across sites/scanners (Supplementary information Figure I-2), it seems safe to assume that the inter-site-variability of the scans is lower than the inter-individual variability of DTI metrics of participants. (2) As in other DTI data-sets, our study was retrospective; thus, scan repetitions (in the event of variable measurement results) were not possible. A major limitation of our *in vivo* neuroimaging approach was the lack of autopsy-confirmed data. Thus, the ROI- and TOI-based analysis can only provide a plausible surrogate *in vivo* “staging” pattern for the presumed PSP pathology in the PSP cohorts investigated here. A further limitation was that an overlap in FA values existed between controls and patients. In future studies, this overlap could be compensated by a large number of controls and subsequent exclusion of control data that lay outside the interquartile range. Due to the intrinsic property of DTI-based FA analysis (Supplementary information III), in order to account for the (slight) overlap of FA values of PSP patients and controls in pattern 3, the decision was made to categorize to CSα when z (< FA >) in pattern 1 was below the threshold of pattern 1 and z (< FA >) in pattern 2 was above the threshold of pattern 2, regardless of z (< FA >) in pattern 3. This constellation was only present in a few PSP patients with z (< FA >) in pattern 3 only slightly above the threshold of pattern 3. Given the character of this *ex post facto* approach, the sequential results in cohort A could not be validated in cohort B because no longitudinal data were available for cohort B. Furthermore, cohort B patients showed a shorter disease duration compared to cohort A. (3) In the current data analysis, we have chosen FA as the appropriate parameter to demonstrate the utility of diffusion-weighted imaging/DTI for the detection of disease-related changes and progression in the brains of PSP patients in terms of imaging-associated neuropathological progression *in vivo*. Further studies might be performed in the future which might substitute FA by other diffusion metrics (e.g., mean diffusivity, radial diffusivity, and axial diffusivity) or tractwise parameters (e.g., fiber density) and could also include volumetric alterations to finally enable a comprehensive AI-based neuroimaging categorization for PSP (Volkmann et al., 2025).

## Conclusion

In conclusion, the novel ROI/TOI-based technique employed here enabled us to analyze specific PSP-associated brain structures categorized into distinct disease steps, thereby rendering it possible to image the proposed neuropathological PSP stages. This procedure constitutes an *in vivo* confirmation of the neuropathological propagation concept of disease stages in PSP. In future applications, it might broaden the spectrum of potential non-invasive surrogate markers as a neuroimaging-based read-out for PSP studies at the group level within a clinical context.

## Data Availability

The data analyzed in this study is subject to the following licenses/restrictions: The dataset used and analyzed during the current study will be made available by the corresponding author upon reasonable request to qualified researchers. Requests to access these datasets should be directed to jan.kassubek@uni-ulm.de.

## Appendix

Members of the *DESCRIBE-PSP* Study Group

Moritz Brandt (Department of Neurology, University Hospital Carl Gustav Carus, Technische Universität Dresden, Dresden, Germany), Katharina Buerger (Institute for Stroke and Dementia Research, University Hospital, LMU Munich, Munich, Germany), Emrah Düzel (Institute of Cognitive Neurology and Dementia Research, Otto-von-Guericke University, Magdeburg, Germany), Björn Falkenburger (Department of Neurology, University Hospital Carl Gustav Carus, Technische Universität Dresden, Dresden, Germany), Agnes Flöel (Department of Neurology, University Medicine Greifswald, Greifswald, Germany), Wenzel Glanz (Clinic for Neurology, Medical Faculty, University Hospital Magdeburg, Magdeburg, Germany), Günter U. Höglinger (Department of Neurology, University Hospital, LMU Munich, Munich, Germany), Daniel Janowitz (Institute for Stroke and Dementia Research, University Hospital, LMU Munich, Munich, Germany), Sabrina Katzdobler (Department of Neurology, University Hospital of Munich, Ludwig-Maximilians-Universität (LMU) Munich, Munich, Germany), Ingo Kilimann (Department of Psychosomatic Medicine, Rostock University Medical Center, Rostock, Germany), Okka Kimmich (Department of Neurology, University of Bonn, Bonn, Germany), Johannes Levin (Department of Neurology, University Hospital of Munich, Ludwig-Maximilians-Universität (LMU) Munich, Munich, Germany), Oliver Peters (Charité – Universitätsmedizin Berlin, corporate member of Freie Universität Berlin and Humboldt-Universität zu Berlin-Institute of Psychiatry and Psychotherapy), Josef Priller (Department of Psychiatry and Psychotherapy, Charité, Berlin, Germany), Johannes Prudlo (Department of Neurology, University Medical Centre, Rostock, Germany), Luisa-Sophie Schneider (Charité – Universitätsmedizin Berlin, corporate member of Freie Universität Berlin and Humboldt-Universität zu Berlin-Institute of Psychiatry and Psychotherapy), Annika Spottke (Department of Neurology, University of Bonn, Bonn, Germany), Eike Jakob Spruth (Department of Psychiatry and Psychotherapy, Charité, Berlin, Germany), Matthis Synofzik (Department of Neurodegenerative Diseases, Hertie Institute for Clinical Brain Research and Center of Neurology, University of Tübingen, Tübingen, Germany), Stefan Teipel (Department of Psychosomatic Medicine, Rostock University Medical Center, Rostock, Germany), Carlo Wilke (Department of Neurodegenerative Diseases, Hertie Institute for Clinical Brain Research and Center of Neurology, University of Tübingen, Tübingen, Germany).
