# Supplementary material for: Diffusion tensor imaging of sequential neuropathological patterns in progressive supranuclear palsy
Source: Front Aging Neurosci. 2025 Jun 6;17:1569302. doi: 10.3389/fnagi.2025.1569302 (PMC12179216; doi:10.3389/fnagi.2025.1569302)
Supplement: Supplementary file 1 [file Data_Sheet_1.pdf]

Supplementary Information I: Subject distribution across centers

In cohort A, data were collected from 12 different centers. All centers (**Supplementary Information Table I-1**) used the identical acquisition protocol for all subjects (see Methods Section, main article). The distribution of participants is shown in **Supplementary Information Figure I-1**.

Supplementary Information Table I-1: *Vendors and models of the MR tomographs at the centers.*

center no.	model	center no.	model
1	Siemens TrioTim	6	Siemens Skyra fit
2	Siemens Verio	7	Siemens TrioTim
3	Siemens TrioTim	8	Siemens Skyra
4	Siemens Skyra	9	Siemens Verio
5	Siemens Biograph_mMR	10	Siemens Prisma fit
		11	Siemens Verio
		12	Siemens Verio

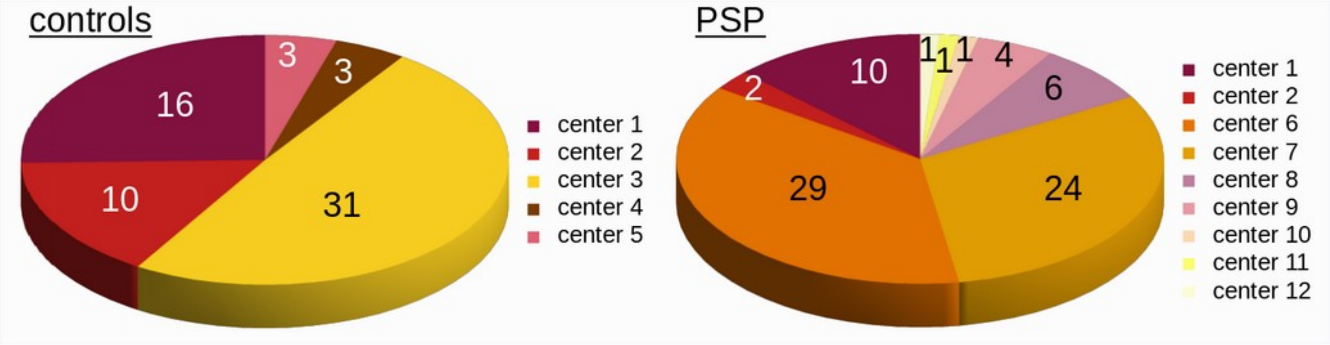

Supplementary Information Figure I-1: *Participants' distribution in centers: 63 controls from 5 different centers (left pie chart), and 78 PSP patients from 9 different centers (right pie chart).*

After MNI normalization, the FA maps of cohort A were tested for variability and deviation which might remain despite the setup of the identical scanning protocol. Average whole brain FA for controls was 0.330 with a standard deviation of 0.016, average whole brain FA for PSP patients was 0.308 witha standard deviation of 0.021, and no outliers were detected in relation to a single center, all values across all sites/scanners were within the range of twice the standard deviation. Furthermore, whole brain FA maps were tested on their correlation to an average FA map; correlation for controls was in average 0.547 with a standard deviation of 0.043, correlation for PSP patients was in average 0.522 with a standard deviation of 0.044, and no outliers were detected in relation to a single center, all values were within the range of twice the standard deviation. A visualisation is provided in **Supplementary Information Figure I-2**.

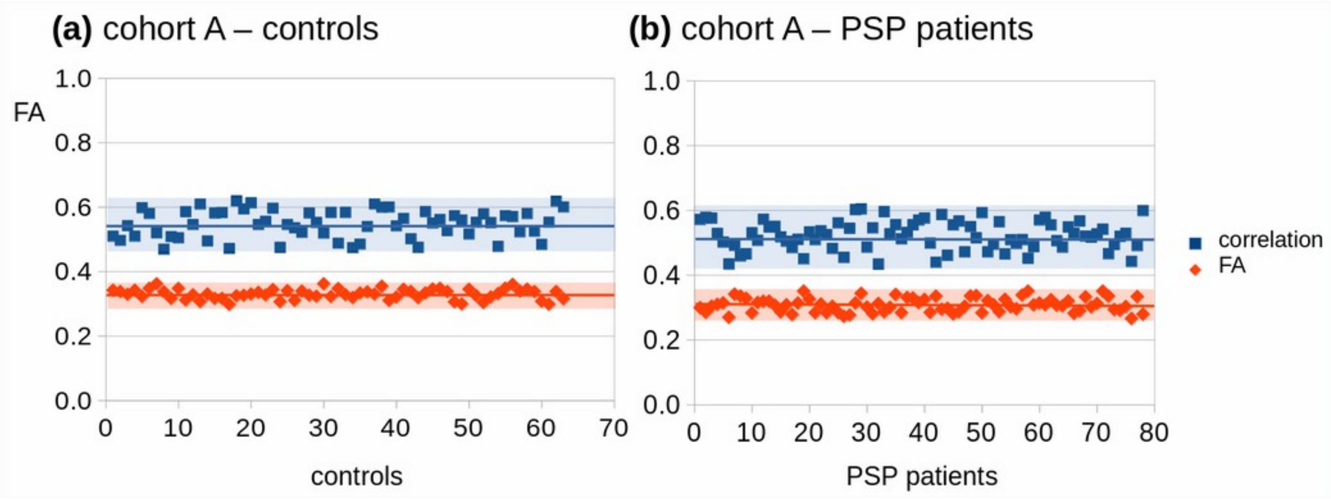

Supplementary Information Figure I-2: *Average whole brain FA for the 63 controls and for the 78 PSP patients of cohort A and correlation to an average FA map. Average of all participants: solid line, ±2*standard deviation: transparency.*

Supplementary Information II. ROIs and TOIs for the three defined patterns

Supplementary Table II-1. Summary of all ROIs and TOIs for the three defined patterns corresponding to sequential involvement during the PSP disease course: *ROI sizes correspond to spherical ROIs with center at the respective MNI coordinate. TOIs (fiber tracts) were reconstructed from an averaged DTI data set (see Figure 1 for the graphical representation); the size of the fiber tracts was provided in voxels covered by the tract (voxel size, 1 mm³). FA values within a given ROI were arithmetically averaged for each subject. The technique of tract-wise fractional anisotropy statistics (TFAS) was applied to quantify the tractography results: by use of the TOI, FA values underlying the tracts were selected for arithmetic averaging. Individual subject´s averaged FA values along each of the above-mentioned tracts were calculated for each tract separately. Student's t-test was used for the statistical comparison of ROI- and tract-based FA values at the group level; significance was defined as $p < 0.05$, corrected for multiple comparisons.*

**Pattern 1**												
**ROI**	pons	cerebral peduncle	midbrain	midbrain tegmentum	putamen/ pallidum							
**MNI**	0/-25/-39	±14/-14/-8	±±/-19/-8	0/-28/-8	±26/-2/3							
**size/voxel**	4169	1419	2109	2109	1419							
**TOI (fiber tract)**	nigrostriatal pathway	medial lemniscus										
**size/voxel**	881	1961										
**Pattern 2**												
**ROI**	caudate	anterior thalamus	frontoorbital WM	prefrontal WM	premotor WM	precentral WM	cerebellar WM	nucleus dentatus				
**MNI**	±14/10/6	±11/-13/3	±20/43/-2	±22/32/22	±29/12/40	±43/-9/39	±30/-66/-30	±15/-57/-29				
**size/voxel**	257	1419	4169	4169	4169	1419	1419	2109				
**TOI (fiber tract)**	anterior thalamic radiation	anterior limb internal capsule	dentato-rubro-thalamic tract	superior cerebellar peduncle	fasciculus fronto-occipitalis	posterior thalamic radiation	fasciculus uncinatus	cortico-striatal pathway	tracts of callosal area I	tracts of callosal area II	tracts of callosal area III	corticospinal tract
**size/voxel**	2498	5139	2442	875	12562	2935	1494	1277	5645	7041	3230	3168
**Pattern 3**												
**ROI**	parietal WM	temporal WM	occipital WM									
**MNI (x/y/z)**	±36/-47/37	±49/-12/-12	±21/-77/7									
**size/voxel**	9171	4169	4169									
**TOI (fiber tract)**	cingulum	fasciculus longitudinalis inferior	fornix	posterior limb internal capsule	tracts of callosal area IV	tracts of callosal area V						
**size/voxel**	1283	4989	854	12582	1371	8857						

Supplementary Information III: Definition of a group separation threshold

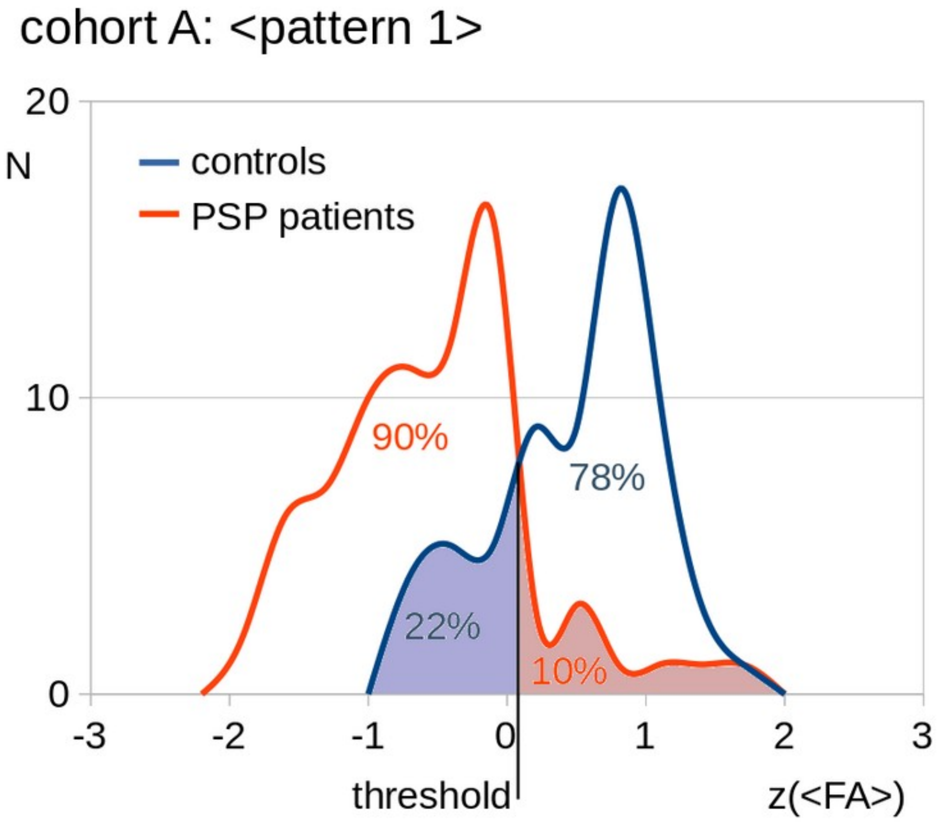

Supplementary Figure III-1: *Histogram for group separation of <FA> in cohort A (pattern 1) for PSP patients vs. controls. The threshold is defined at a specificity of 78% (N=49 out of 63).*

Supplementary Information IV. Whole brain-based statistics (WBSS) results of FA maps

Supplementary Table IV-1: WBSS results of FA maps. *MNI – Montreal Neurological Institute.*

cl. no.	no. of voxels	MNI (x/y/z)	p (average)		anatomical localization
**Cohort A: WBSS at baseline: 78 PSP patients vs. 63 controls**					
1	310521	0 / -28 / -27	< 0.0001	FA decrease	extended alteration throughout the WM
2	20001	-21 / 2 / -2	< 0.0004	FA increase	putamen L
3	998	24 / 2 / 2	< 0.0004	FA increase	putamen R
**Cohort B: WBSS at baseline: 66 PSP patients vs. 44 controls**					
1	299024	0 / -24 / -29	< 0.0001	FA decrease	extended alteration throughout the WM
2	1343	26 / -3 / 1	< 0.0002	FA increase	putamen R
3	1212	-22 / 3 / -3	< 0.0002	FA increase	putamen L
**Cohort A: WBSS 21 PSP patients (with follow-up scan) at baseline vs. 63 controls**					
1	231871	0 / -18 / -29	< 0.0003	FA decrease	extended alteration throughout the WM
2	1636	-23 / 1 / -2	< 0.0004	FA increase	putamen L
3	812	24 / 3 / 2	< 0.001	FA increase	putamen R
**Cohort A: WBSS 21 PSP patients at follow-up vs. 63 controls**					
1	314756	0 / -18 / -30	< 0.0001	FA decrease	extended alteration throughout the WM
2	1804	-22 / 2 / -2	< 0.001	FA increase	putamen L
3	855	24 / 1 / -1	< 0.001	FA increase	putamen R
**Cohort A: WBSS at baseline: 23 PSP-P patients vs. 44 controls**					
1	108504	0 / -28 / -27	< 0.001	FA decrease	extended alteration throughout the WM
2	33188	-41 / 30 / 7	< 0.001	FA decrease	frontal lobe
3	6279	-38 / -29 / 6	< 0.001	FA decrease	temporal lobe
**Cohort A: WBSS at baseline: 55 PSP-RS patients vs. 44 controls**					
1	277201	0 / -28 / -27	< 0.001	FA decrease	extended alteration throughout the WM
2	1532	-21 / 2 / -2	< 0.001	FA increase	putamen L
3	972	24 / 2 / 2	< 0.001	FA increase	putamen R
**Cohort B: WBSS at baseline: 20 PSP-P patients vs. 44 controls**					
1	180818	0 / -28 / -27	< 0.001	FA decrease	extended alteration throughout the WM
2	1448	45 / -2 / 21	< 0.001	FA decrease	temporal lobe
3	1167	24 / 2 / 2	< 0.001	FA increase	putamen R
4	823	-21 / 2 / -2	< 0.001	FA increase	putamen L
**Cohort B: WBSS at baseline: 46 PSP-RS patients vs. 44 controls**					
1	282966	0 / -28 / -27	< 0.001	FA decrease	extended alteration throughout the WM
2	1092	24 / 2 / 2	< 0.001	FA increase	putamen R
3	957	-21 / 2 / -2	< 0.001	FA increase	putamen L

Supplementary Information V. Differences of <FA> for ROIs and TOIs between PSP patients and controls

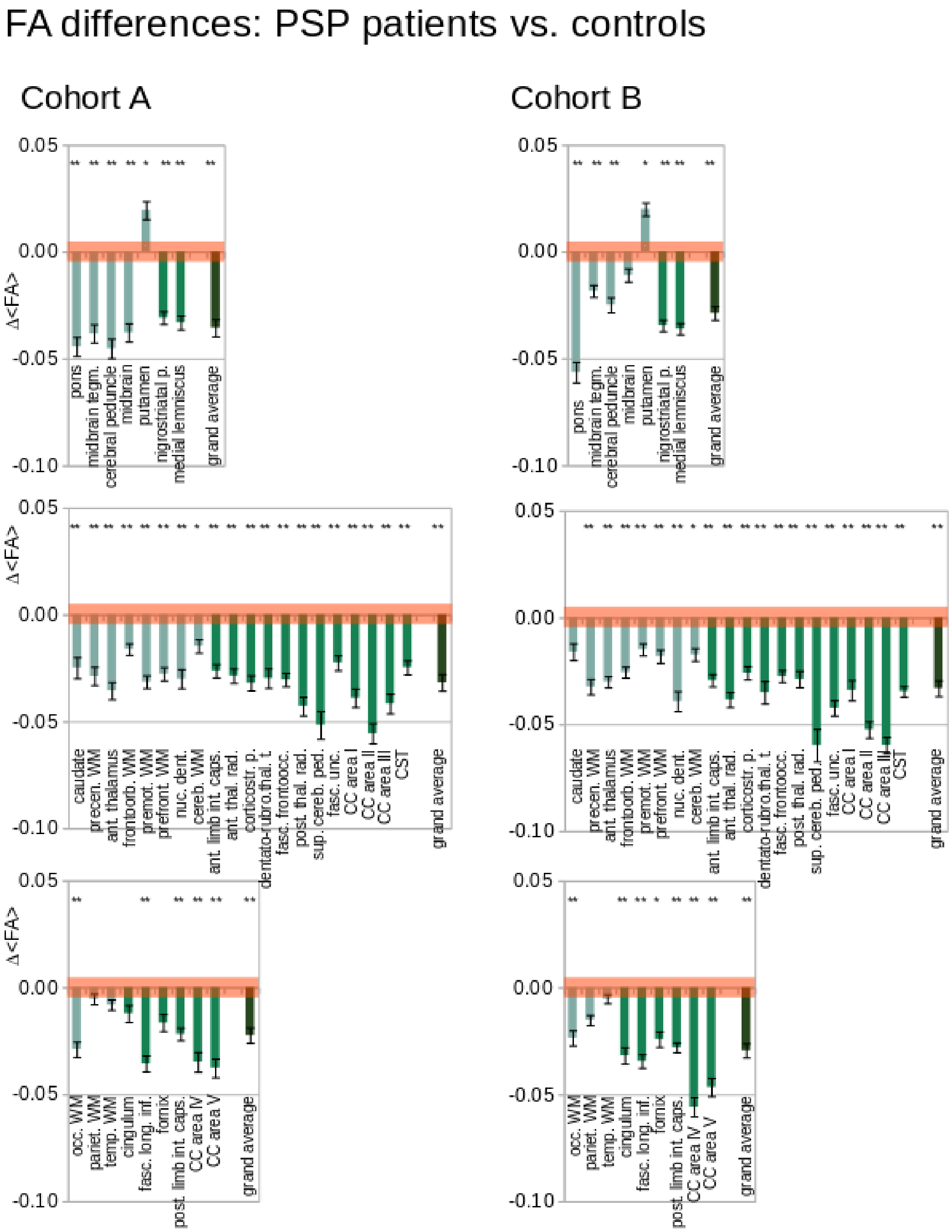

Supplementary Figure V-1. Differences of <FA> for ROIs and TOIs between PSP patients and controls at baseline for cohort A (left) and B (right). Upper panel: *pattern 1-related structures;* ***middle panel:*** *pattern 2-related structures;* ***lower panel:*** *pattern 3-related structures. The transparent orange interval indicates an error estimation obtained from baseline and follow-up controls' scans. * p < 0.01; ** p < 0.0001, corrected for multiple comparisons.*

Supplementary Information VI. Individual examples of longitudinal alterations of z<FA> for the three patterns.

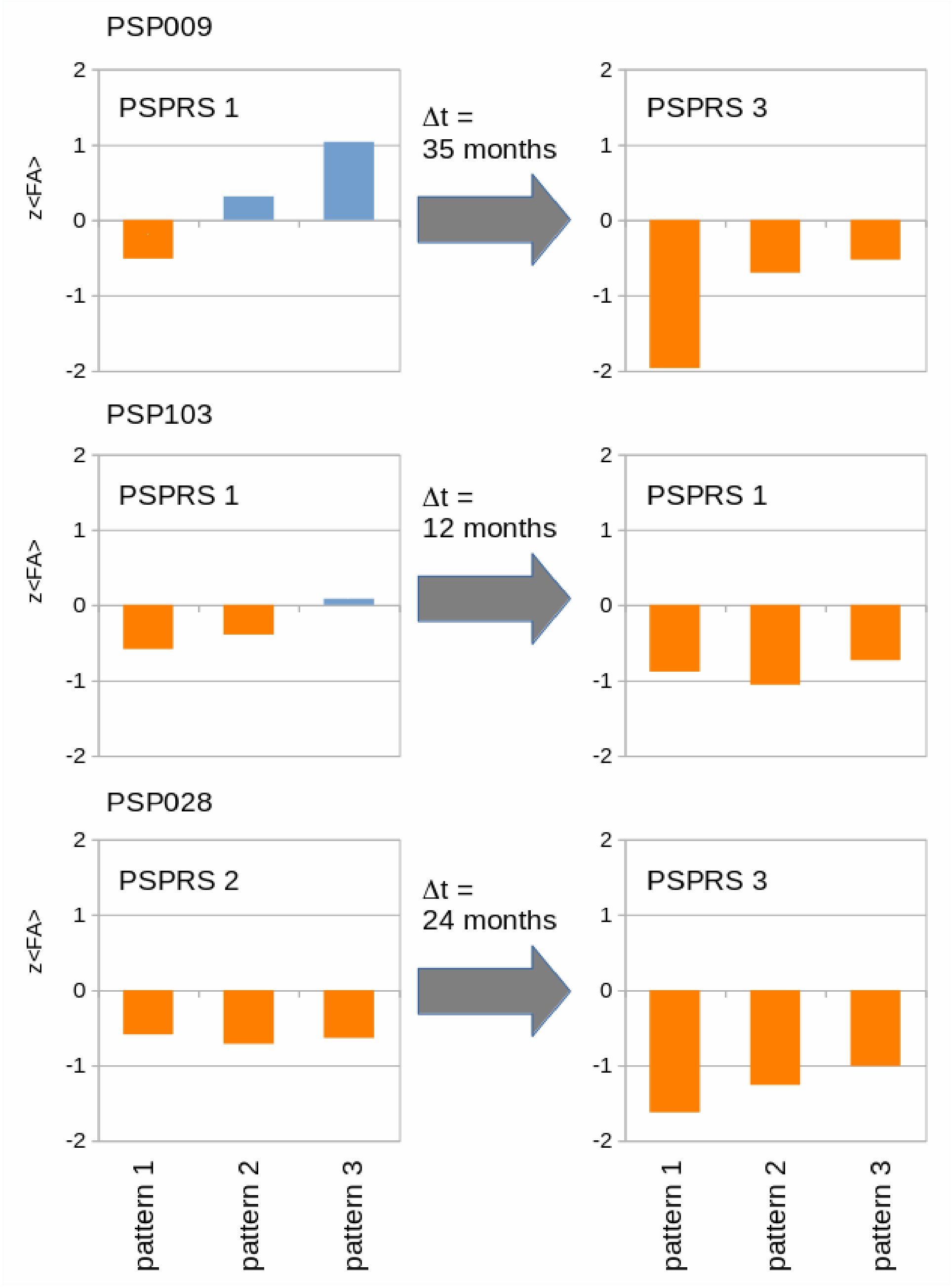

Supplementary Figure VI-1: *Individual examples of longitudinal alterations of z<FA> for the three patterns.*

Supplementary Information VII. Cross-sectional comparison of PSP-RS patients vs. controls and PSP-P vs. controls at baseline and at follow-up

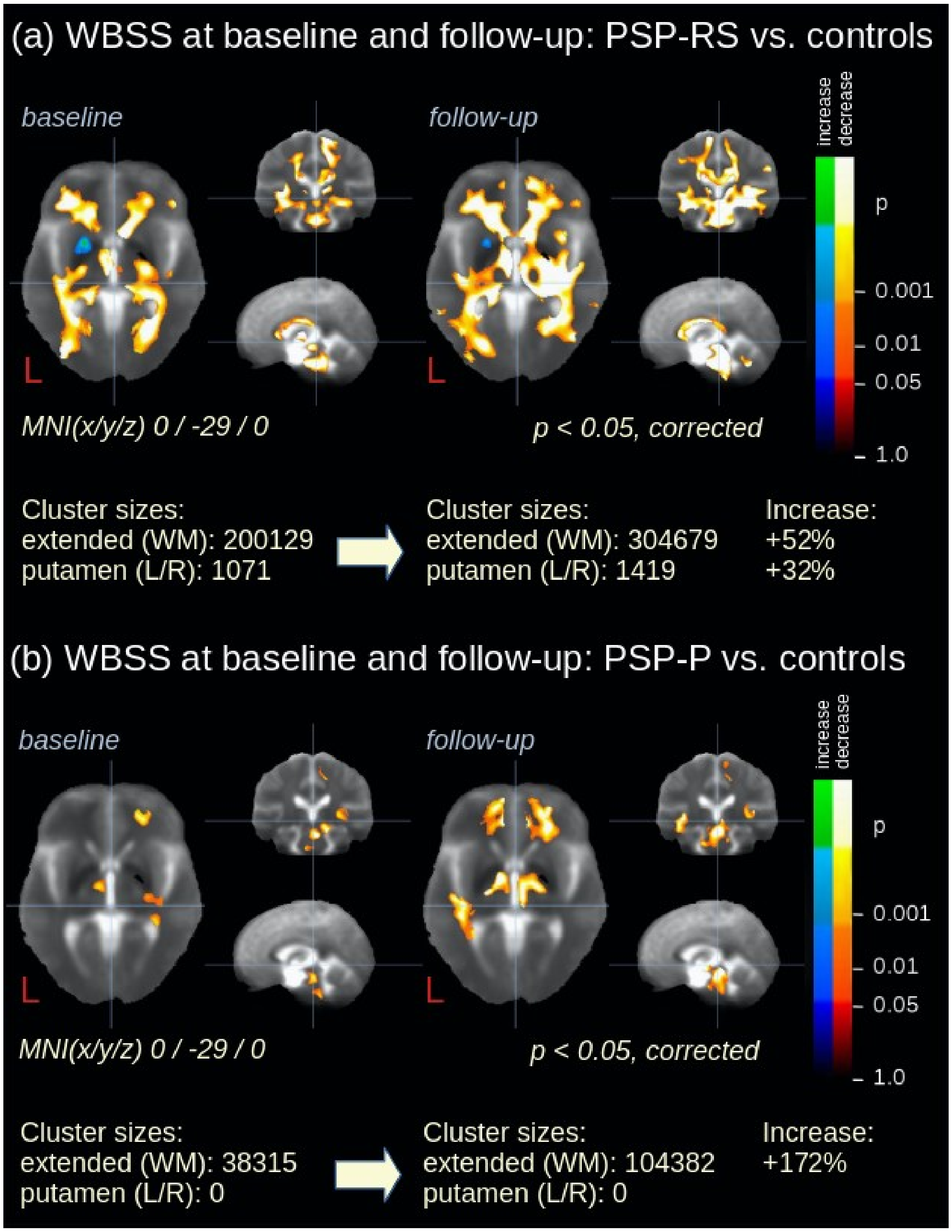

Supplementary Figure VII-1: Whole brain-based spatial statistics (WBSS) in subtypes PSP with Richardson's syndrome (PSP-RS – a) and predominant parkinsonism (PSP-P – b). *Thirteen PSP-RS vs. controls showeds more extended significant FA decrease compared to 8 PSP-P vs. controls at baseline; at follow-up, both groups showed expanded clusters (see cluster sizes / sum of voxels). Due to statistical reasons (small subject numbers), affectation clusters (FA increase) in the bilateral putamen for PSP-P and in the right putamen for PSP-RS were below the detection threshold. MNI – Montreal Neurological Institute coordinate frame.*

Supplementary Information VIII. Percentage of PSP patients into imaging-based categorization steps

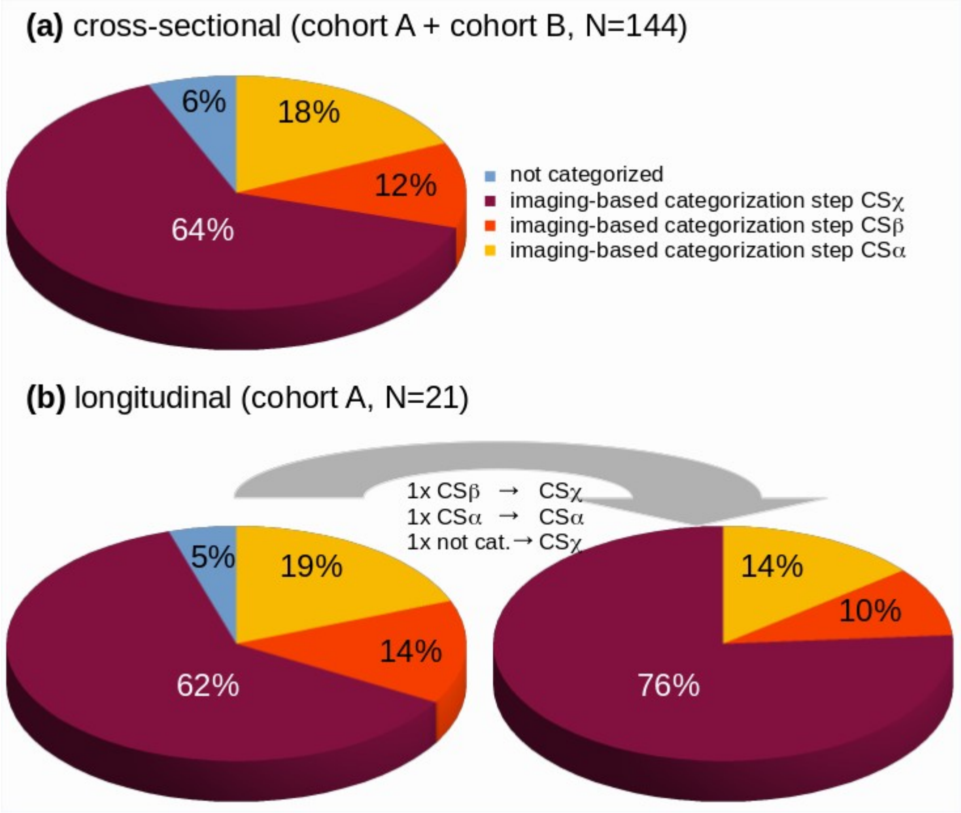

Supplementary Figure VIII-1. *Percentage categorization of PSP patients into imaging-based categorization steps CSα, CSβ, and CSχ (and not categorized) for (a) the baseline samples (cohort A and cohort B, N = 144) as well as for (b) the longitudinal sample (cohort A, N = 21).*

Supplementary Information IX. Effect size and sample size calculations

Standardised effect size calculations for the different patterns 1, 2, and 3 (see Table 3-II) were performed by

$$E = (z(<\Delta FA>_{controls} – z(<\Delta FA>_{PSP}) / \sigma_{controls,PSP} \quad .$$

The sample size calculations for future clinical studies were performed based on a two-sided significance level of 5% (group mean difference with $p<0.05$) and a power of 80%; then, a good approximation to calculate the minimum sample size S from the effect size E for a desired treatment effect t is given by

$$S > 15.7/(E * t)^2 + 0.96$$

SupplementaryTable IX-1*: Effect sizes and sample sizes for the patterns 1, 2, and 3.*

	**pattern 1**	**pattern 2**	**pattern 3**
**E**	1.68	1.32	0.71
**S**	5	9	30

Supplementary Information X. Cross-sectional comparison of PSP patients vs. controls according to PSP Rating Scale (PSPRS) stages

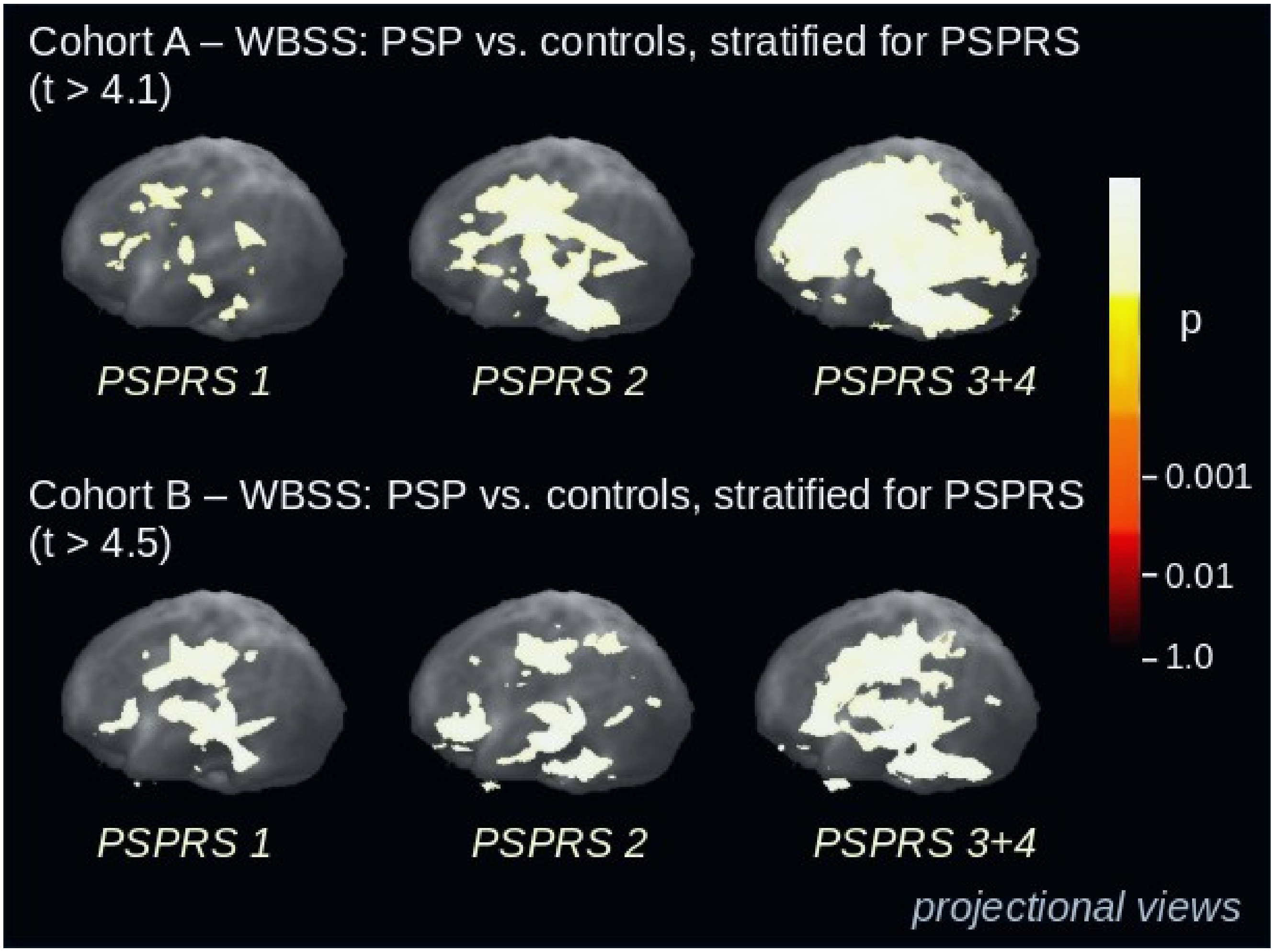

***Supplementary Figure X-1: Whole brain-based statistics (WBSS) (cross-sectional comparison) of groups of PSP patients vs. controls according to PSPRS stages for cohorts A (upper panel) and B (lower panel)** – projectional sagittal views.*
